# The Combination of Buprenorphine and Pregabalin in the Management of Peripheral Neuropathic Pain: A Phase IV, Randomized, Double‐Blind and Placebo‐Controlled Clinical Trial

**DOI:** 10.1002/ejp.70359

**Published:** 2026-08-17

**Authors:** Larissa I. Moreira, Pedro N. Martins, Manoel J. Teixeira, Daniel C. de Andrade, Gabriel T. Kubota

**Affiliations:** ^1^ Pain Center, Department of Neurology University of São Paulo São Paulo Brazil; ^2^ Federal University of Juiz de Fora Juiz de Fora Brazil; ^3^ Center for Neuroplasticity and Pain, Department of Health Sciences and Technology, Faculty of Medicine Aalborg University Aalborg Denmark

**Keywords:** buprenorphine, chronic pain, neuralgia, opioid analgesics, pregabalin

## Abstract

**Background:**

Pharmacological combinations are commonly used for treating refractory neuropathic pain, although supporting evidence is lacking. Gabapentinoid‐opioid combination is one of the most studied; however, their efficacy is inconsistent across clinical trials. This trial investigated the feasibility, effectiveness and safety of adding pregabalin to buprenorphine for managing this condition.

**Methods:**

This single‐center, phase IV, double‐blind, placebo‐controlled trial enrolled adults with peripheral chronic neuropathic pain, refractory to tricyclic antidepressants and/or serotonin‐norepinephrine reuptake inhibitors. Participants were randomized to receive transdermal buprenorphine up to 20 mcg/day, plus pregabalin up to 300 mg/day (group C) or placebo (group M). Participants underwent a 3‐week tolerability‐based dose titration and a 6‐week maintenance phase. Primary outcome was ≥ 30% average pain reduction at week 9.

**Results:**

Sixty individuals (66.7% female, 53 [45–58.5] years old) were enrolled. After titration, pregabalin dose was 100 mg/day for most group C participants (87%), and transdermal buprenorphine doses were similar between groups (*p* = 0.787). At week 9, ≥ 30% pain reduction was achieved by 38% in group C and 32% in group M (*p* = 0.942). Pain interference, mood, anxiety and quality‐of‐life scores did not significantly differ between groups. Baseline pain phenotype was not associated with primary outcome results. Side‐effect frequencies were similar between groups, and no serious adverse events occurred.

**Conclusions:**

Adding pregabalin to transdermal buprenorphine was not found to be effective for treating neuropathic pain. However, low‐to‐moderate analgesic effects cannot be excluded. Although no major side effects occurred, opioid use may have led to lower pregabalin tolerability, hindering the feasibility of this combination in clinical practice.

**Significance Statement:**

Although pharmacological combinations are widely prescribed for neuropathic pain (NP), consistent evidence of their benefit is lacking. This phase IV, pragmatic trial does not support the effectiveness of pregabalin‐buprenorphine combination for refractory NP, even across distinct pain phenotypes. Besides suggesting the poor feasibility of this combination due to poor tolerability, it adds to the mounting evidence challenging earlier reports of an opioid‐sparing effect for gabapentinoids.

Clinical Study Registration Number: 52145215.0.0000.0068.

## 1 Introduction

Chronic neuropathic pain (NP) is a debilitating condition that is estimated to affect 7%–10% of the worldwide population (van Hecke et al. 2014). Several pharmacological classes have been found to be efficacious for this condition and are recommended by current guidelines (Soliman et al. 2025), but its treatment remains challenging. When using first‐ and second‐line drugs, only about a quarter of patients achieve clinically significant pain relief (Moisset et al. 2022; Moulin et al. 2015). Indeed, when used in monotherapy, the average effect size of these pharmacological treatments is moderate at best (Soliman et al. 2025). Moreover, although the long‐term benefit and safety of opioids for NP treatment remain uncertain (Bialas et al. 2020), due to the suboptimal results of other therapeutical alternatives, about a third of these individuals are prescribed these medications (Moisset et al. 2022; Moulin et al. 2015), and they are recommended as third‐line options for its management (Soliman et al. 2025).

One reason that may explain this refractoriness is that NP may present with distinct pain mechanisms across individuals (Finnerup et al. 2020). Therefore, it is improbable that any single medication would have its action mechanisms matching the pain mechanisms in all patients. This rationale supports the combination of medications with differing mechanisms of action to improve the likelihood of pain control. However, there are still relatively few data exploring the clinical utility of combining medications for treating NP (Balanaser et al. 2023), particularly in those refractory to the most effective first‐line options: tricyclic antidepressants and venlafaxine/duloxetine (Soliman et al. 2025). Notably, one of the most investigated combinations are of opioids and gabapentinoids; even though data regarding the benefit of such approach are inconsistent when compared to opioid monotherapy (Balanaser et al. 2023). Furthermore, few opioids have been explored in these studies, mostly being typical ones, that is, morphine or oxycodone (Balanaser et al. 2023).

Particularly, to the best of our knowledge, the effectiveness of pharmacological combinations including buprenorphine for NP has not been assessed so far. Buprenorphine acts as both a mu‐opioid receptor agonist and a kappa‐opioid receptor antagonist, reducing adverse events rates and overdose risk when compared to typical opioids (Evans and Easthope 2003). It has been shown to be efficacious for treating chronic non‐cancer pain in clinical trials with follow‐ups lasting over 12 weeks (Wong et al. 2023); and its interesting safety profile has contributed to its approval by the FDA for treating opioid use disorder (Kumar et al. 2025). These characteristics make it an interesting alternative when opioid therapy is considered for treatment of NP refractory to first‐ and second‐line therapies. Moreover, combining it with gabapentinoids may optimize analgesic outcomes while reducing the need for higher opioid doses.

This clinical trial aimed at investigating whether buprenorphine and pregabalin combination is feasible, safe and could offer superior analgesic benefit than buprenorphine alone for people suffering from peripheral NP refractory to antidepressants. With this, we seek to determine whether additive effects exist between these medications that can offer superior pain relief and improved outcomes for NP, while reducing the need for higher opioid doses to achieve these benefits, thereby partially mitigating the long‐term risks associated with opioid therapy.

## 2 Methods

### 2.1 Study Design

This was a Phase IV, randomized, double‐blind, placebo‐controlled, parallel‐arms pragmatic clinical trial, which aimed to assess the effectiveness and safety of the combination therapy with buprenorphine and pregabalin, compared to buprenorphine alone, for treating refractory peripheral NP. Individuals with peripheral NP due to polyneuropathy, refractory to at least one other first‐line medication for treatment of this condition (i.e., tricyclic antidepressant or serotonin‐norepinephrine reuptake inhibitor) were randomized in a 1:1 ratio to receiving either transdermal buprenorphine with pregabalin (group C), or transdermal buprenorphine and matching placebo capsules (group M). After screening (baseline visit, V0), enrolment and baseline assessment (visit 1, V1), participants underwent a 3‐week flexible titration period (visits 2 to 4, V2 to V4); and were then followed during an additional 6‐week maintenance phase (visits 5 and 6, V5 and V6). While intermediate evaluations during titration (V2 and V3) and maintenance (V5) periods were made by phone, baseline (V1) and the remaining assessments (V4 and V6) were conducted in‐person.

**FIGURE 1:**
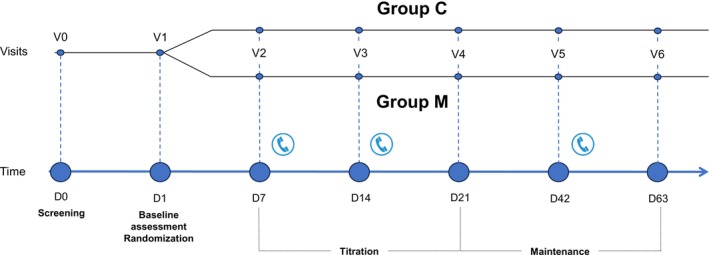
Study design. This figure presents the general design of this clinical trial. The phone image indicates the visits which were conducted by phone, and the remaining were in‐person assessments. Group C received transdermal buprenorphine and pregabalin, while Group M received transdermal buprenorphine monotherapy.

Pain intensity and side‐effects were assessed in all visits, while quality‐of‐life, mood, anxiety and pain interference scores were evaluated in V0, V4 and V5 (Figure 1).

This trial was conducted at an outpatient clinic of the Pain Center of the Neurology Department at the Hospital das Clínicas of the University of São Paulo Medical School, Brazil. It received approval from our Institution's Review Board (approval no. 1.433.857) before the inclusion of the first participant and was registered online since the 2nd of March, 2016. All subjects provided informed consent before enrollment.

### 2.2 Participants

This study included individuals with definite chronic peripheral NP due to polyneuropathy, irrespective of its aetiology, according to the following criteria: (i) ≥ 18 years‐old; (ii) suffering from NP, defined as a score ≥ 4 on the Douleur Neuropathique 4 (DN4) questionnaire (Bouhassira et al. 2005; Santos et al. 2010); (iii) pain duration of at least 3 months; (iv) average pain intensity of ≥ 4 on the visual analog scale (VAS), despite the use of other first‐line medication for NP, supported by current guidelines (i.e., tricyclic antidepressant and/or serotonin‐norepinephrine reuptake inhibitor) (Soliman et al. 2025); (v) able to understand and cooperate with questionnaires as well as provide informed consent. Subjects were excluded from this study, in case of: (i) previous use of or contraindication to pregabalin or buprenorphine; (ii) current use of other α‐2‐δ ligand or other opioids; (iii) pregnant or lactating women; (iv) history of allergic reaction or other contraindication to the use of transdermal patches; (v) having participated in another clinical trial within 28 days of randomization; (vi) having undergone any modification in the current treatment for neuropathic pain in the past 3 months.

### 2.3 Blinding and Randomization

Participants were allocated to either the combined therapy (group C) or the monotherapy group (group M) at a 1:1 ratio, according to a randomized computer‐generated list produced by an open software, made available at www.randomizer.org (Copyright 1997–2010 by Geoffrey C. Urbaniak and Scott Plous). Group allocation was masked to both the participants and researchers responsible for outcome assessments and statistical data analyses, throughout the duration of the study. The study medications were dispensed by a pharmacist in packages identified with the participant's study number, and containing instructions on how to use them. Placebo capsules were indistinguishable from pregabalin capsules in terms of physical aspects (i.e., weight, size and colour). At the final study visit (V6), a blinding assessment was performed, according to a previously reported protocol (Rocha et al. 2014). It consisted of questioning all participants: (1) if they were able to tell which medications they received; and, if so, (2) which it was.

### 2.4 Study Interventions

Treatment was initiated with 5 mcg/h of buprenorphine (for both groups), administered through a transdermal patch which was changed weekly, as well as 50 mg pregabalin capsules (group C) or matching placebo (group M) taken every 12 h. During the three‐week titration period, participants were monitored weekly for side effects, initially by telephone (V2 and V3), and finally in person (V4). At each visit, they were instructed to progressively increase the total daily study medication dosage up to a maximum of 20 mcg/day for transdermal buprenorphine and 300 mg/day for pregabalin. In case clinically significant side effects were reported on any of these visits, they were advised to reduce the dose to the highest previously tolerated. These individuals were then instructed to maintain the achieved medication dosage for another 6 weeks (maintenance phase), at the end of which they were again assessed in person (V6). Throughout this trial, participants were instructed to continue any medications already in use at baseline for NP treatment, without any dose changes. Also, they were not allowed to start any different treatment for this condition for the duration of this study. To verify treatment adherence, study medication patches and capsules were counted at every in‐person visit.

### 2.5 Outcomes and Clinical Assessments

The primary outcome of this study was the proportion of subjects in each group who achieved a ≥ 30% reduction in their average pain intensity, when compared to baseline (i.e., 30% responders) at V6. Pain intensity was measured at every study visit with a visual analog scale (VAS), anchored at 0 (no pain) and 100 mm (worst possible pain). Other pain characteristics, as well as its interference in the individual functionality were evaluated with the brief pain inventory (BPI) (Cleeland and Ryan 1994; Ferreira et al. 2011) and the neuropathic pain symptoms inventory (NPSI) (de Andrade et al. 2011; Bouhassira et al. 2004) at V1, V4 and V6. Furthermore, at these three visits, mood and anxiety symptoms were investigated with the hospital anxiety and depression scale (HADS) (Botega et al. 1995; Zigmond and Snaith 1983); and at V1 and V6 quality‐of‐life was examined with the WHOQoL‐BREF questionnaire (Fleck et al. 2000; The WHOQOL Group 1998). Data collection, storage and management were conducted using REDCap (Nashville, TN, EUA) electronic capture tools hosted at University of Sao Paulo Medical School (Harris et al. 2009, 2019).

### 2.6 Adverse Effects Assessment

The occurrence of adverse events was investigated at all study visits, with a structured questionnaire containing all side effects described in the study medications labels, as well as by spontaneous participant report. An adverse event was considered to be related to the research when it occurred after the initial dose of the study medication. Adverse events were classified as severe or non‐severe. Severe adverse events were defined as those which resulted in hospitalization, death or long‐term sequelae.

### 2.7 Statistical Methods

To the best of our knowledge, no study has previously assessed the effectiveness of combined pregabalin and buprenorphine therapy for NP. Therefore, it was calculated that a sample size of 50 subjects would be necessary to assess the difference in the proportion of 30% responders between the study groups, at a Cohen's h of 0.8, with a statistical power of 80%, and assuming an α error probability of 0.05. Considering a drop‐out rate of 20%, this study aimed to achieve a total sample size of 60 individuals. Sample size calculation was conducted with the software G*Power 3.1.9.7 (Faul et al. 2007).

Baseline clinical and demographic characteristics were described using relative and absolute frequencies, when categorical, and medians and interquartile ranges, when continuous. The primary outcome (≥ 30% reduction in the VAS for average pain intensity) was compared between the treatment and placebo groups using the chi‐square test. For that purpose, a modified intention‐to‐treat analysis was performed, including only the participants who attended at least one titration visit after randomization (i.e., received at least one pack of treatment). Missing values were handled using multiple imputation, with treatment arm and all outcome assessments as the supporting variables. Predictive mean matching was used within the mice package with five iterations. To better understand the changes in VAS over time, we conducted an exploratory analysis using a mixed model for repeated measures with the interaction between time (visit) and treatment group, considering the random intercept according to the patient.

Secondary outcomes were compared between groups using the chi‐square test when categorical, and their changes between the first and last visits were compared using the Wilcoxon test when continuous. No adjustments for baseline variables were prespecified in the statistical analysis plan. However, a post hoc sensitivity analysis was conducted by repeating the multiply‐imputed mixed model analysis for VAS pain intensity adjusted for the variables found to differ significantly between groups at baseline. Significance was set at *p* = 0.05.

### 2.8 Prespecified Subgroup Analyses

NP is a complex phenomenon, which encompasses several distinct underlying mechanisms, that may reflect in its clinical presentation (Campbell and Meyer 2006). Therefore, it is possible to hypothesize that specific clinical features, or clusters of characteristics, of this type of pain may indicate differing biological underpinnings and consequently predict treatment responses (Finnerup et al. 2020). This concept has been supported by previously published *post hoc* analyses from clinical trials with both pharmacological and non‐pharmacological therapies for NP, which indicated that clinical features captured by the NPSI can be associated with a higher probability of therapeutic success (Bouhassira et al. 2021; da Cunha et al. 2022; Freeman et al. 2014; Min et al. 2021). In particular, the researchers' clinical experience suggests that a non‐continuous evoked pain phenotype may indicate a better response to combined buprenorphine and pregabalin treatment, compared to buprenorphine monotherapy. To test this secondary hypothesis, a planned prespecified subgroup analysis was conducted. For that, participants were classified in two groups, according to the NPSI scores, as follows: (i) Predominantly spontaneous and continuous, if the total score of the items assessing spontaneous and continuous pain (items 1, 2 and 3) was > 15 points; (ii) Predominantly non‐spontaneous and non‐continuous, if this score was ≤ 15 points. Finally, another planned prespecified analysis was also performed to investigate whether the NPSI clinical phenotypes, previously identified in the abovementioned studies (Bouhassira et al. 2021; Freeman et al. 2014), would also be associated with therapy response. For these analyses, logistic regression models were constructed to investigate the heterogeneity of effect according to the subgroups of interest.

## 3 Results

### 3.1 Data Collection and Sample Characteristics

**FIGURE 2:**
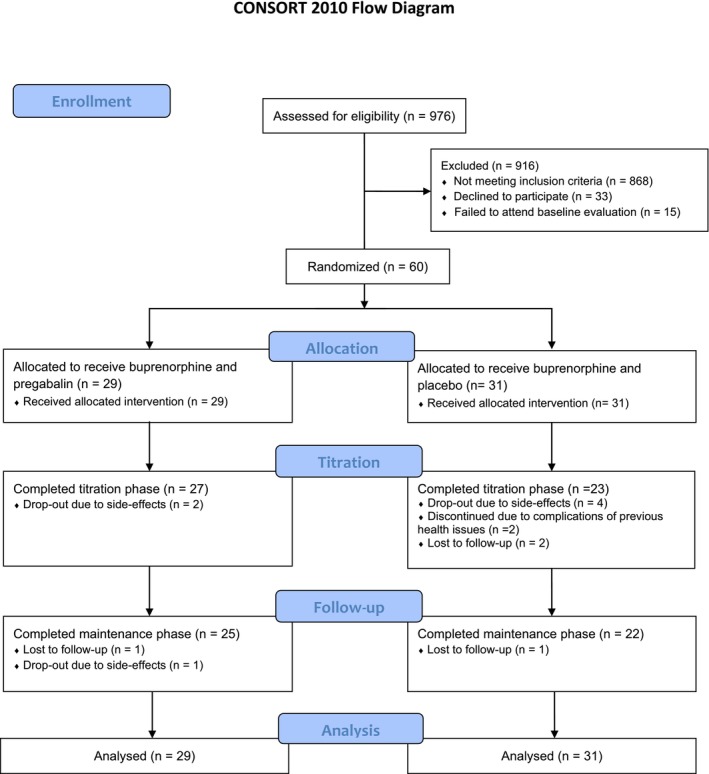
CONSORT flow diagram for data collection.

Enrollment occurred from August 2017 to September 2021. In total, 976 subjects were screened for eligibility, and 60 of them were included in this trial (Figure 2). Their median age was 53 (45–58.5) years and 66.7% were female. Half of the participants (*n* = 30) were randomized to group C and the other half (*n* = 30) to group M. Anxiety levels (*p* = 0.041), pain interference (*p* = 0.046), total (*p* = 0.047) and paroxysmal pain (*p* = 0.037) NPSI scores were significantly higher among individuals in Group M. Although the median pain duration at enrollment was numerically smaller (6.5 [4.0–10.8] vs. 10.0 [5.0–15.0] years) and the frequency of systemic hypertension (51.7% vs. 36.7%) and major depressive disorder (51.7% vs. 36.7%) were numerically larger in group M, these differences were not statistically significant (*p* > 0.05 for all). The remaining baseline characteristics were similar between groups (Table 1). A total of 47 individuals completed the follow‐up, and the attrition rate was similar between groups C and M (13.7% vs. 29.0%, *p* = 0.152).

**TABLE 1:** General demographic features of the study participants.

	Group C (*N* = 29)	Group M (*N* = 31)	*p*
Age (years‐old)^a^	54 (40–61)	52.5 (48.5–57.0)	0.994
Female sex	19 (66)	20 (68)	0.855
Ethnicity			
White	14 (48)	17 (55)	0.277
Afro descendant	13 (45)	14 (45)	
Asian	2 (7)	0 (0.0)	
Pain duration (years)^a^	11 (6–16)	6 (4–10)	0.136
Comorbidities			
Major Depression Disorder	11 (38)	15 (50)	0.351
Systemic hypertension	10 (34)	16 (53)	0.145
Diabetes Mellitus	5 (17)	4 (13)	0.731
Alcoholism	4 (15)	3 (10)	0.696
VAS average pain intensity^a^	7.0 (6.0–8.0)	8.0 (7.0–8.0)	0.453
Brief Pain Inventory^a^			
Pain severity index	7 (6–8)	7 (6–8)	0.458
Pain interference index	5 (4–9)	8 (6–9)	0.046*
DN4 score^a^	8 (7–9)	7 (6–9)	0.095
NPSI total score^a^	25 (18–32)	30 (22–36)	0.047*
Burning spontaneous pain subscore	8 (5–9)	7 (6–9)	0.881
Pressing spontaneous pain subscore	5 (3–6)	5 (4–8)	0.269
Paroxysmal pain subscore	4 (0–6)	5 (3–9)	0.037*
Evoked pain subscore	4 (3–7)	6 (4–8)	0.055
Paresthesia/dysesthesia subscore	6 (4–8)	7 (4–9)	0.128
NPSI cluster^b^			
Pinpointed	8 (29)	4 (13)	0.339
Deep	9 (32)	13 (43)	
Evoked	11 (39)	13 (43)	
Pain clinical phenotype			
Predominantly continuous and spontaneous	15 (52)	17 (55)	0.809
Predominantly non‐continuous and non‐spontaneous	14 (45)	14 (48)	
HADS total score^a^	16 (11–21)	21 (15–27)	0.090
Anxiety subscore	7 (5–10)	10 (8–14)	0.041*
Depression subscore	9 (6–13)	10 (8–13)	0.423
WHOQoL‐BREF^a^			
Overall quality‐of‐life assessment	5 (4–7)	6 (5–7)	0.218
Physica domain	18 (16–21)	17 (14–20)	0.218
Psychological domain	20 (16–22)	18 (13–22)	0.353
Social relationships domain	11 (9–12)	10 (8–12)	0.257
Environment domain	25 (22–27)	23 (19–26)	0.092

*Note:* Group C—combined transdermal buprenorphine and pregabalin treatment; Group M—transdermal buprenorphine monotherapy; Y—defined as a total score of > 15 points in items 1, 2 and 3 of the NPSI; Z—defined as a total score of ≤ 15 points in items 1, 2 and 3 of the NPSI; Data presented as *n* (%), unless otherwise specified.

Abbreviations: DN4, Douleur Neuropathique‐4 Questionnaire; HADS, Hospital Anxiety and Depression Scale; NPSI, Neuropathic Pain Symptoms Inventory; VAS, visual analog scale.

^a^ Data presented as median (interquartile range).

^b^ Defined according to the work of Freeman et al. (2014).

* *p* < 0.05.

### 3.2 Primary and Other Pain‐Related Outcomes

At the end of the maintenance phase (V6), the proportion of responders was not significantly different between groups C and M (41% vs. 39%, OR = 1.13 [95% CI 0.32–3.99]; *p* = 0.942), nor were the average pain intensity VAS scores between them (7.0 [4.0–8.0] vs. 5.0 [4.0–8.0]; *p* = 0.636) (Table 2). Notably, these findings remained unchanged after adjusting the analysis for baseline variables found to differ significantly between groups (Table S5). The trajectory of these VAS scores remained similar between groups throughout all intermediate follow‐up evaluations (*p* > 0.05 for all interactions between time and group; Table S1 and Table S2). Moreover, no significant differences could be observed between them in terms of BPI severity (*p* = 0.776) and interference indexes (*p* = 0.364). DN4 (*p* = 0.809) and NPSI total scores (*p* = 0.863) and subscores (*p* > 0.05 for all) were also similar between groups C and M.

**TABLE 2:** Primary and secondary study outcomes according to treatment group at the end of the maintenance phase.

	End of maintenance phase (V5)		Change from baseline		*p*
	Group M (*n* = 31)	Group C (*N* = 29)	Group M (*n* = 31)	Group C (*N* = 29)	
≥ 30% reduction in VAS average pain intensity^a^	7 (32)	9 (38)	—	—	0.942
VAS average pain intensity	5 (4 to 8)	7 (4 to 8)	1 (0 to 3)	2 (−1 to 4)	0.636
Brief Pain Inventory			—	—	
Pain severity index	6 (4 to 7)	6 (3 to 7)	1 (0 to 3)	2 (0 to 4)	0.776
≥ 50% reduction in pain severity index	2 (9)	6 (27)	—	—	0.554
Pain interference index	5 (3 to 7)	3 (2 to 8)	2 (1 to 5)	2 (0, 4)	0.364
≥ 50% reduction in pain interference index	6 (29)	9 (38)	—	—	0.815
DN4 total score	6 (5 to 9)	5 (3 to 7)	1 (−1 to 3)	1 (0 to 3)	0.809
NPSI total score	23 (14 to 29)	16 (11 to 19)	7 (0 to 16)	11 (4 to 12)	0.863
Burning spontaneous pain subscore	6 (3 to 7)	5 (0 to 7)	1 (0 to 3)	2 (0 to 5)	0.292
Pressing spontaneous pain subscore	3 (0 to 4)	3 (0 to 4)	2 (0 to 4)	2 (0 to 5)	> 0.999
Paroxysmal pain subscore	6 (3 to 8)	1 (0 to 4)	1 (−1 to 4)	1 (0 to 4)	0.848
Evoked pain subscore	4 (2 to 6)	3 (1 to 4)	1 (−1 to 3)	1 (0 to 3)	0.712
Paresthesia/dysesthesia subscore	5 (4 to 7)	4 (3 to 6)	2 (−1 to 4)	1 (0 to 3)	> 0.999
HADS total score	16 (12 to 22)	9 (5 to 24)	2 (−1 to 6)	7 (−2 to 10)	0.815
Anxiety subscore	9 (4 to 11)	5 (2 to 12)	2 (−1 to 5)	3 (0 to 6)	0.785
Depression subscore	9 (5 to 12)	6 (3 to 11)	0 (−2 to 6)	3 (0 to 6)	0.398
WHOQoL‐BREF					
Overall quality‐of‐life assessment	6 (5 to 7)	6 (5 to 8)	0 (−1 to 0)	0 (−1 to 1)	0.504
Physica domain	20 (16 to 23)	21 (16 to 23)	−2 (−4 to 2)	−2 (−5 to 1)	0.761
Psychological domain	19 (16 to 21)	20 (17 to 22)	0 (−1 to 1)	−1 (−3 to 1)	0.671
Social relationships domain	10 (8 to 12)	11 (9 to 12)	0 (−1 to 2)	0 (−1 to 1)	0.633
Environment domain	23 (20 to 26)	24 (21 to 25)	1 (−1 to 2)	1 (−1 to 3)	0.585

*Note:* Group C—combined transdermal buprenorphine and pregabalin treatment; Group M—transdermal buprenorphine monotherapy; Y—defined as a total score of > 15 points in items 1, 2 and 3 of the NPSI; Z—defined as a total score of ≤ 15 points in items 1, 2 and 3 of the NPSI; Data presented as median (interquartile range), unless otherwise specified.

Abbreviations: DN4, Douleur Neuropathique‐4 Questionnaire; HADS, Hospital Anxiety and Depression Scale; NPSI, Neuropathic Pain Symptoms Inventory; VAS, visual analog scale.

^a^ Data presented as *n* (%).

### 3.3 Subgroup Analyses According to Phenotype

There was no association between baseline NP clinical phenotypes (i.e., predominantly continuous and spontaneous vs. predominantly non‐continuous and non‐spontaneous) and the probability to achieve a ≥ 30% reduction in average pain intensity VAS at the end of the maintenance phase (*p* = 0.545). Also, no interaction between baseline NPSI clusters and the primary outcome was observed (Table S3).

### 3.4 Other Secondary Outcomes

No significant differences were found between groups C and M at the end of follow‐up (V5) for total HADS scores (*p* = 0.815), nor its anxiety (*p* = 0.785) and depression subscores (*p* = 0.398). Overall quality‐of‐life assessments (*p* = 0.504) were similar between treatment groups, as well as all WHOQoL‐BREF domains scores (Table 2). After titration, most participants from group C received 100 mg/day (87%) of pregabalin (Table S4). Moreover, daily buprenorphine doses were similar between groups C and M (*p* = 0.787), as in both most individuals achieved either 5 mcg/day (52% vs. 56%, respectively) or 10 mcg/day (39% vs. 44%, respectively).

### 3.5 Blinding Assessment

There was no significant difference between the proportion of subjects who reported being able to guess which treatment they received between groups C and M (54% vs. 61.5%, *p* > 0.999). Also, among these individuals, the proportion of subjects who guessed their group allocation correctly was not significantly different between these two groups (group C = 40% vs. group M = 23%, *p* = 0.193; Table S6).

### 3.6 Adverse Events

The most reported adverse event throughout the study was xerostomia (66.6%), followed by constipation (61.6%) and pruritus at the site of the transdermal patch (53.3%). Headache was significantly more frequent among individuals receiving buprenorphine monotherapy, when compared to those being treated with both buprenorphine and pregabalin (47% vs. 21%, *p* = 0.043*, Table 3). No other reported adverse effects differed significantly between groups. Overall, seven individuals (11.6%) dropped out due to side‐effects, four in group M and three in group C (*p* = 0.757). No serious adverse effects occurred. Two individuals from group M discontinued due to complications of previous stroke and cancer, respectively, which were deemed not related the studied medications.

**TABLE 3:** Adverse events reported during study follow‐up.

	Group M	Group C	*p*
	(*N* = 31)	(*N* = 29)	
Xerostomia	21 (72)	19 (70)	0.866
Constipation	21 (70)	16 (57)	0.309
Pruritus at the site of the transdermal patch	14 (47)	18 (62)	0.235
Nausea	18 (60)	12 (41)	0.153
Dizziness	13 (43)	17 (59)	0.240
Drowsiness	16 (53)	12 (43)	0.425
Headache	14 (47)	6 (21)	0.043*
Flatulence	6 (21)	8 (31)	0.392
Lethargy	9 (31)	6 (23)	0.508
Vomiting	9 (31)	10 (36)	0.708
Blurred vision	5 (17)	6 (23)	0.589
Increased appetite	6 (21)	6 (23)	0.831
Confusion	7 (24)	5 (19)	0.660
Irritability	6 (21)	3 (12)	0.475
Euphoria	3 (10)	3 (12)	> 0.999

* *p* < 0.05.

## 4 Discussion

This was the first Phase IV, pragmatic, randomized, double‐blind, placebo‐controlled clinical trial to have assessed the effectiveness, feasibility and safety of adding pregabalin to transdermal buprenorphine for treating otherwise refractory peripheral NP. This combination was not found to be more effective when compared to buprenorphine monotherapy in improving neuropathic pain. Furthermore, there was no indication that it led to improvements in mood, anxiety or quality‐of‐life scores. These findings were maintained even after prespecified subgroup analyses, according to NP clinical phenotypes. Notably, although no major adverse events were observed, pregabalin dose titration was restricted due to frequent side effects, suggesting limited feasibility of this combined pharmacological approach in clinical practice.

Given the relatively small number of trials that have explored combined pharmacotherapy for NP treatment (Balanaser et al. 2023), this strategy is not supported by current guidelines (Soliman et al. 2025). Nevertheless, because NP is frequently refractory to available first‐line medications, over half of people suffering from this condition are prescribed two or more analgesic drugs (Berger et al. 2012; Harrisson et al. 2020). Notably, among the pharmacological class combinations to have been previously explored in clinical trials, the addition of opioids and gabapentinoids is one of the most studied (Balanaser et al. 2023). Interest in this combination may have been fueled by initial studies indicating a potential opioid‐sparing effect of short‐term gabapentinoid use in the context of acute perioperative analgesia (Ho et al. 2006). Although this concept has been questioned by posteriorly published trials and remains controversial (Verret et al. 2020), research with animal and in vitro models has provided possible biological mechanisms that may support this effect. For example, recently, gabapentinoids have been demonstrated to promote the cyclic AMP inhibiting effect of opioids by reducing the calcium influx through L‐type voltage gated calcium channels (Garza‐Carbajal et al. 2024). Moreover, gabapentin has been suggested to enhance the anti‐nociceptive effect of morphine by activating the IL‐10‐HO‐1 signalling pathway (Bao et al. 2014).

Despite this initial promising evidence, results from clinical trials exploring opioid and gabapentinoids combination for chronic NP have been inconsistent (Balanaser et al. 2023). Two short duration trials (i.e., less than 8 weeks) demonstrated a larger benefit with the addition of gabapentin when compared to placebo, to ongoing morphine (Gilron et al. 2005) or any opioid treatment (Caraceni et al. 2004). Contrastingly, another cross‐over trial with short treatment duration (2 weeks) did not observe significant differences in pain intensity scores when adding pregabalin versus placebo to morphine treatment, although it did observe a significant reduction in the minimal effective morphine dose after the combination (Dou et al. 2017). Moreover, in line with the results of the present study, the largest available trial also did not observe superiority of combined pregabalin 300 mg/day with tapentadol 300 mg/day, when compared to tapentadol 500 mg/day monotherapy for 8 weeks, among individuals who had failed a three‐week course of tapentadol 300 mg/day (Baron et al. 2015). On the other hand, evidence arising from two clinical trials consistently indicated that the addition of oxycodone to gabapentinoid treatment seems to improve analgesic results when compared to gabapentinoid monotherapy (Balanaser et al. 2023).

Several hypotheses may explain these inconsistencies. For example, it is possible that an eventual beneficial effect of such combination may not be generalizable to all gabapentinoids nor all opioids. Indeed, previous trials with promising results have addressed the association of gabapentin to an opioid (usually, morphine) (Caraceni et al. 2004; Gilron et al. 2005). And, although the initial evidence spanning up to the mid‐2010's has suggested that both gabapentin and pregabalin have similar effectiveness in treating neuropathic pain (Finnerup et al. 2015), recently published studies have put this into question. In fact, the mean analgesic effect size found in trials assessing the effectiveness of pregabalin for treating NP has dropped significantly when studies published between 2001 and 2005 are compared to those in 2016 and 2020 periods (Cheng et al. 2023). Furthermore, in a head‐to‐head cross‐over trial, Robertson et al. (2019) observed that not only did gabapentin provide larger pain relief than pregabalin in individuals suffering from sciatica, but it also resulted in significantly less side effects. Another aspect that may explain the more promising results with the combination of gabapentin with opioids, rather than pregabalin, is that morphine has been shown to increase the plasma concentration of gabapentin among healthy individuals (Eckhardt et al. 2000). This finding has been postulated to be the result of enhancements in intestinal gabapentin absorption, due to opioid‐induced decrease in gut mobility. Since oral bioavailability of pregabalin is much higher than gabapentin and not dose‐dependent, it may not benefit from this interaction mechanism (Eckhardt et al. 2000). While it is also true that gabapentinoids association may not lead to additional benefit to all opioid therapies for NP, most research has only assessed morphine, oxycodone and tapentadol and, to the best of our knowledge, no previously published study has investigated buprenorphine (Balanaser et al. 2023; Baron et al. 2015).

Moreover, it is possible that only specific subsets of NP subjects may benefit from the combination of gabapentinoid and opioids. This is supported by research with other pharmacological and non‐pharmacological NP therapies, which suggested that the reported clinical features of this type of pain (i.e., clinical phenotype) may predict treatment response (Bouhassira et al. 2021; Freeman et al. 2014). While this possibility has not been supported by the results of the prespecified subgroup analyses of the data from this trial, they may have been underpowered by its relatively small sample. Moreover, there may be other clinical aspects, that could identify a subset of individuals with higher probability of treatment response, which were not investigated here. Particularly, an ad hoc analysis of the data from the previously mentioned trial which assessed the effectiveness of pregabalin and tapentadol combination (Baron et al. 2015), has found that baseline sleep disorders may predict better results with this combination, when compared to tapentadol monotherapy (Otto et al. 2019). Unfortunately, sleep quality was not explored in this trial.

Although this trial did not observe an additional benefit in combining pregabalin to buprenorphine therapy for NP, this association seemed generally safe for up to 9 weeks. As with other studies with buprenorphine and pregabalin, side effects were common (Derry et al. 2019; Wong et al. 2023). However, no severe adverse event was observed and drop‐out due to side effects were similar between groups. Moreover, the incidence of these adverse reactions was similar between pregabalin/buprenorphine combination and buprenorphine monotherapy groups. Nonetheless, it should be noted that most individuals in the combination group were only able to tolerate the initial 100 mg/day dose of pregabalin during the titration phase. While this may have undermined the effectiveness of the treatment, it also may suggest a lower tolerability to pregabalin‐induced side‐effects among individuals using opioids. This association has been reported in a previously published meta‐analysis including observational studies, particularly for central nervous system side‐effects, that is, dizziness, cognitive disfunction and respiratory depression (Hahn et al. 2022). Notably, when this analysis was performed among randomized clinical trials, this association was not observed (Hahn et al. 2022). This highlights the role of pragmatic trials, such as this, in overcoming the limitations of trials with traditional designs in reflecting the feasibility and effectiveness of interventions in real‐world practice (Hohenschurz‐Schmidt et al. 2024). Opioid‐gabapentinoid combination has also been reported to be associated with higher rates of substance use disorder and opioid‐related deaths (Gomes et al. 2017; Muller et al. 2024). Additionally, it should be noted that the risk for significant side effects increases with long‐term opioid therapy (Bialas et al. 2020; Noble et al. 2010), and as most trials addressing the use opioid for NP, this study had a follow‐up of less than 12 months. Also, in animal models, the threshold for opioid‐induced respiratory depression has been found to decrease when gabapentinoids are administered (Tambon et al. 2023). While the risk for this serious adverse event may be reduced with buprenorphine when compared to other strong opioids, due to its characteristic bell‐shaped dose–response curve for side‐effects (Lizasoain et al. 1991), caution is advised when high doses are used and with at‐risk patient groups, circumstances which were both not addressed in the present trial.

It should be highlighted that the results of our trial should be viewed, taking into account its limitations. Our study was single‐centered and only included individuals with peripheral NP due to polyneuropathy refractory to other first‐line medications; therefore, its results may not be generalizable to other populations. Nonetheless, polyneuropathy remains the most common cause of NP (van Hecke et al. 2014), and opioid therapy is currently only recommended to individuals refractory to other treatment alternatives (Soliman et al. 2025). Given the scarcity of prior data to inform sample size calculations for this opioid–gabapentinoid combination trial, and considering the aforementioned risks associated with this combination, sample size was estimated assuming a large effect size (Faul et al. 2007), reflecting the threshold of benefit that would justify its use in clinical practice. Consequently, low‐to‐moderate benefits cannot be excluded. However, considering the abovementioned recent evidence indicating significant concerns for long‐term opioid‐gabapentinoid combinations, the use of this association would be more appropriately justified in case of larger benefits. Additionally, as this study compared the pregabalin–buprenorphine combination with buprenorphine monotherapy, no conclusions can be drawn regarding the effectiveness and safety of this combination compared to pregabalin monotherapy. Data about the aetiology of polyneuropathy was not obtained, which might limit interpretation of the generalizability of the study results. Nonetheless, current approaches recommended for pain management are mechanism‐ rather than aetiology‐based (Soliman et al. 2025; Vardeh et al. 2016). The attrition rate was also larger than expected, and the total number of individuals who completed the follow‐up was somewhat smaller than the estimated required sample size, which may have underpowered the effectiveness analyses. As mentioned above, this may have also reduced the sensitivity of the prespecified subgroups analyses for identifying individuals with higher response probability, according to the NP phenotype. Furthermore, although there was no clear indication that data were not missing at random, the possibility of attrition bias cannot be excluded. Missing data was addressed in our analyses with multiple imputation, which is a widely used and recommended approach (Hopewell et al. 2025; Sterne et al. 2009). However, the validity of this method still depends on the assumption that data were missing at random and on correct specification of the imputation model, either of which, if violated, could introduce residual bias into the treatment effect estimates. Finally, it should be highlighted that most participants in the combination therapy group have not achieved the minimally therapeutic dose ≥ 150 mg/day of pregabalin, and this may have contributed significantly to the negative findings of this study. Notably, however, most trials with pregabalin have been conducted among predominantly Caucasian western and Asian individuals (Soliman et al. 2025), and it is possible that its therapeutic dosing range, as well as its tolerability, may vary in other populations, due to genetic‐related pharmacodynamic reasons.

In conclusion, this study did not observe any additional analgesic benefit of the addition of pregabalin to buprenorphine therapy for peripheral NP. Also, no subset of individuals with higher probability of response to this combined therapy could be identified when the clinical pain phenotype was considered. Although no serious adverse events were observed, concomitant opioid use may have reduced pregabalin tolerability, limiting dose titration and the effectiveness of this drug combination. Additionally, low‐to‐moderate analgesic effects cannot be presently excluded. Therefore, this trial suggests that the reticences towards the use of combination therapy for NP in general can also be extended to refractory NP in particular.

## Author Contributions

This study was designed by D.C.A. and data collection was performed by L.I.M. Data was analysed and interpreted by P.N.M., M.J.T., D.C.A. and G.T.K. The draft of the original manuscript was produced by L.I.M., P.N.M., and G.T.K. All authors have reviewed the manuscript critically and approved it in its final version, and agree to be accountable for all aspects of this work.

## Funding

This work was supported and funded by LIM62—Pain Center of the Department of Neurology, at the University of Sao Paulo Medical School Clinics Hospital. Mundipharma Brasil Produtos Médicos e Farmacêuticos Ltda provided the transdermal buprenorphine patches, as well as the placebo and pregabalin capsules, under an Investigator Initiated Research Initiative. However, it had no other in the initiative to conduct the trial, its design, data collection, data analyses, drafting of the manuscript, revision of the manuscript, nor in the decision on where and when to publish it. It also never had access to the data of the study before its publication.

## Disclosure

Use of Artificial Intelligence: No artificial intelligence was used.

## Conflicts of Interest

The authors declare no conflicts of interest.

## Supporting information


**Table S1:** Average pain intensity visual analogue scores throughout the study visits.
**Table S2:** Mixed model for pain intensity visual analog scale scores throughout the study follow‐up.
**Table S3:** Logistic model intercations for ≥ 30% average pain intensity reduction according neuropathic pain phenotype.
**Table S4:** Total daily dose of study medications at the end of titration phase.
**Table S5:** Pooled treatment effect on pain intensity visual analog scale scores at each assessment visit, adjusted for baseline pain interference index, NPSI total score, and HADS Anxiety subscore.
**Table S6:** Blinding assessment.

## Data Availability

Anonymized data not published within this article will be made available upon reasonable request from any qualified investigator.
