# Supplementary material for: The Combination of Buprenorphine and Pregabalin in the Management of Peripheral Neuropathic Pain: A Phase IV, Randomized, Double‐Blind and Placebo‐Controlled Clinical Trial
Source: Eur J Pain. 2026 Aug 17;30(7):e70359. doi: 10.1002/ejp.70359 (PMC13479043; doi:10.1002/ejp.70359)
Supplement: Supplementary file 1 [file EJP-30-0-s001.docx]

**Table S1. Average pain intensity visual analogue scores throughout the study visits.**

|  | **Group M**  (n = 31) | **Group C**  (n = 29) |
| --- | --- | --- |
| **Visit 1** | 8.0 (7.0, 8.0) | 7.0 (6.0, 8.0) |
| **Visit 2** | 7.0 (5.0, 8.0) | 6.0 (5.0, 8.0) |
| **Visit 3** | 7.0 (4.0, 8.0) | 6.0 (5.0, 7.0) |
| **Visit 4** | 5.0 (3.0, 8.0) | 6.0 (3.0, 8.0) |
| **Visit 5** | 5.0 (2.0, 8.0) | 6.0 (3.0, 8.0) |
| **Visit 6** | 5.0 (3.0, 9.0) | 7.0 (3.0, 8.0) |

*Data presented as median (Q1, Q3)*

**Table S2. Mixed model for pain intensity visual analogue scale scores throughout the study follow-up.**

|  | **Beta** **(95%CI)** | **p** |
| --- | --- | --- |
| **Group** |  |  |
| *M* | — |  |
| C | .23 (-11, 15) | 0.729 |
| **Follow-up assessments** |  |  |
| *Visit 1* | — |  |
| *Visit 2* | -9.5 (-23, -3.8) | 0.153 |
| *Visit 3* | -12 (-24, 0.54) | 0.061 |
| *Visit 4* | -14 (-31, -3.7) | 0.111 |
| *Visit 5* | -20 (-38, -2.4) | 0.029* |
| *Visit 6* | -17 (-37, 4.2) | 0.104 |
| **Interaction Group * Visit** |  |  |
| *Treatment * Visit 2* | -0.41 (-15, 16) | 0.957 |
| *Treatment * Visit 3* | -1.9 (-21, 17) | 0.838 |
| *Treatment * Visit 4* | -7.1 (-23, 9.3) | 0.393 |
| *Treatment * Visit 5* | -13 (-38, 12) | 0.272 |
| *Treatment * Visit 6* | -2.7 (-25, 19) | 0.794 |

*95% CI – 95% Confidence Interval; Group M – transdermal buprenorphine monotherapy; Group C - transdermal buprenorphine and pregabalin combined therapy*

** p<0.05*

*** p<0.001*

**Table S3. Logistic model intercations for ≥ 30% average pain intensity reduction according neuropathic pain phenotype.**

| **Variable** | **ROR**^A^ | **95%CI**^B^ | **p-value** |
| --- | --- | --- | --- |
| Phenotype Interactions (Ref.: Y) |  |  |  |
| Group C * Z | 0.50 | 0.05, 5.02 | 0.545 |
| Cluster Interactions (Ref.: Pinpointed) |  |  |  |
| Group C * Deep | 2.04 | 0.07, 62.1 | 0.677 |
| Group C * Provoked | 0.45 | 0.01, 21.8 | 0.675 |
| *ROR = Ratio of Odds Ratios; 95%CI = 95% Confidence Interval* | | |  |

**Table S4. Total daily dose of study medications at the end of titration phase**

|  | **Group C** (n = 23) | **Group M** (n = 27) | **p** |
| --- | --- | --- | --- |
| **Pregabalin (mg/day)** |  |  | --- |
| *100* | 20 (87) | --- |  |
| *200* | 2 (8.7) | --- |  |
| *300* | 1 (4.3) | --- |  |
| **Buprenorphine (mg/week)** |  |  | 0.787 |
| *5* | 12 (52) | 15 (56) |  |
| *10* | 9 (39) | 12 (44) |  |
| *20* | 2 (8.7) | 0 (0) |  |
| Data presented as n (%) | | |  |

| **Table S5. Pooled treatment effect on pain intensity visual analogue scale scores at each assessment visit, adjusted for baseline pain interference index, NPSI total score, and HADS Anxiety subscore.** | | | |
| --- | --- | --- | --- |
| Visit | Mean difference | 95% CI | p |
| Visit 1 | 0.02 | -1.33; 1.37 | 0.975 |
| Visit 2 | -0.10 | -1.51; 1.31 | 0.888 |
| Visit 3 | -0.09 | -1.67; 1.50 | 0.914 |
| Visit 4 | 0.59 | -1.38; 2.56 | 0.538 |
| Visit 5 | 0.73 | -1.52; 2.97 | 0.496 |
| Visit 6 | 0.40 | -1.82; 2.62 | 0.704 |

NPSI – Neuropathic Pain Symptom Inventory; HADS – Hospital Anxiety and Depression Scale

| **Table S6. Blinding assessment** | | | |
| --- | --- | --- | --- |
|  | **Group C**  (n = 26) | **Group M**  (n = 26) | **p** |
| **Subjects who reported being able to guess which treatment they received** | 14 (54%) | 16 (61.5%) | >0.999 |
| **Treatment allocation guessed^A^** |  |  | 0.874 |
| *Buprenorphine monotherapy* | 4 (16%) | 6 (37.5%) |  |
| *Buprenorphine-pregabalin combination* | 10 (40%) | 10 (62.5%) |  |

*Data presented as n (%)*

^A^ Proportions from the subset of participants who reported being able to guess their treatment allocation.
